# Supplementary material for: Rspo2 inhibits TCF3 phosphorylation to antagonize Wnt signaling during vertebrate anteroposterior axis specification
Source: Sci Rep. 2021 Jun 28;11:13433. doi: 10.1038/s41598-021-92824-6 (PMC8239024; doi:10.1038/s41598-021-92824-6)
Supplement: Supplementary file 1 — Supplementary Information. [file 41598_2021_92824_MOESM1_ESM.pdf]

Rspo2 inhibits TCF3 phosphorylation to antagonize Wnt signaling during vertebrate anteroposterior axis specification.

Alice H. Reis and Sergei Y. Sokol

Supplementary Information:

Supplementary Figure legends

Supplementary Fig. 1: Genes upregulated by Rspo2 in ectoderm explants. Top gene targets encoding transcription factors differentially expressed at stage 10 are shown. Four-cell embryos were injected with 0.5 ng of Rspo2 RNA into animal blastomeres. Ectoderm explants were prepared from injected embryos at stage 9, RNA was isolated at stage 10 for RNA-sequencing. Human gene symbols are shown, except for those that are *Xenopus*-specific (*). Target genes of the injected animal caps have been normalized to the uninjected controls.

Supplementary Fig. 2: Rspo2 constructs with compromised binding of ZNRF3/RNF43 and LGR4/5 retain the anteriorizing activity. A, Point mutant constructs generated from Rspo2 and Rspo∆T. B-E, Cement gland enlargement (dashed white line) caused by Rspo2 RNA injection. Ventral animal blastomeres of four-cell embryos were injected with 500 pg of Rspo2, R65A, Q70A, F105A or F109A mRNA. G-I, Anteriorized phenotypes caused by injection of Rspo∆T, Rspo∆T-R65A, Rspo∆T-Q70A, Rspo∆T-F105A∆T or Rspo∆T-F109A mRNA (500 pg each). Imaging was done at stages 28-32. F, M, Quantification of the

embryos showing enlarged cement gland after overexpression of different Rspo2 constructs. Numbers of embryos per group are shown on the top of each bar. N, levels of expression for Rspo2 and different RspoΔT derivatives in the same experiment. Lysates from the injected embryos at stages 11/12 were immunoblotted and probed with anti-Flag antibody. ERK serves as a control for loading.

Supplementary Fig. 3: Rspo2 inhibits Wnt target genes in ectoderm explants. Wnt target gene expression in ectoderm explants at stage 13. Embryos were injected into four animal blastomeres with Wnt8 DNA (50 pg) and Rspo2 RNA (0.5 ng). RT-qPCR analysis was carried out in triplicates for *myod1* and *msgn1*.

Supplementary Fig. 4. Comparison of the anteriorizing activity of Rspo2 constructs. Ventral animal blastomeres of four-cell embryos were injected with 500 pg of RNA encoding Rspo2 deletion mutants. Embryo images, representative of three independent experiments, are shown. The ratio of the number of anteriorized embryos to the total number of injected embryos is indicated. A, Control embryo at stage 27. B-D, the sibling embryos injected with RNAs encoding full-length Rspo2 (B), RspoΔF (C), RspoΔT (D).

Supplementary Tables 1-2.

Supplementary Table 1. Quantification of data for wholemount in situ

hybridization shown in representative images of Figure 1E-J. Numbers of embryos with indicated changes in gene expression are indicated.

Supplementary Table 2. Primer sequences for the site-directed mutagenesis and RT-qPCR.

Suppl. Fig.1

Top genes upregulated by Rspo2		
Human symbol	log2(Fold_change) normalized	p-value
OTX2	5,20	1,0882E-147
TCF24	4,70	0,00023719
CRX/otx5*	3,98	2,7623E-183
GBX2	3,80	0,010773101
ZIC3	3,61	4,00229E-35
NKX2-8	3,46	0,002037888
KLF5	3,46	0,002037888
hes3.L*	3,32	0,040847184
hes5.1.L*	3,32	0,003825822
NFKB1	3,32	0,040847184

Suppl.Fig.2

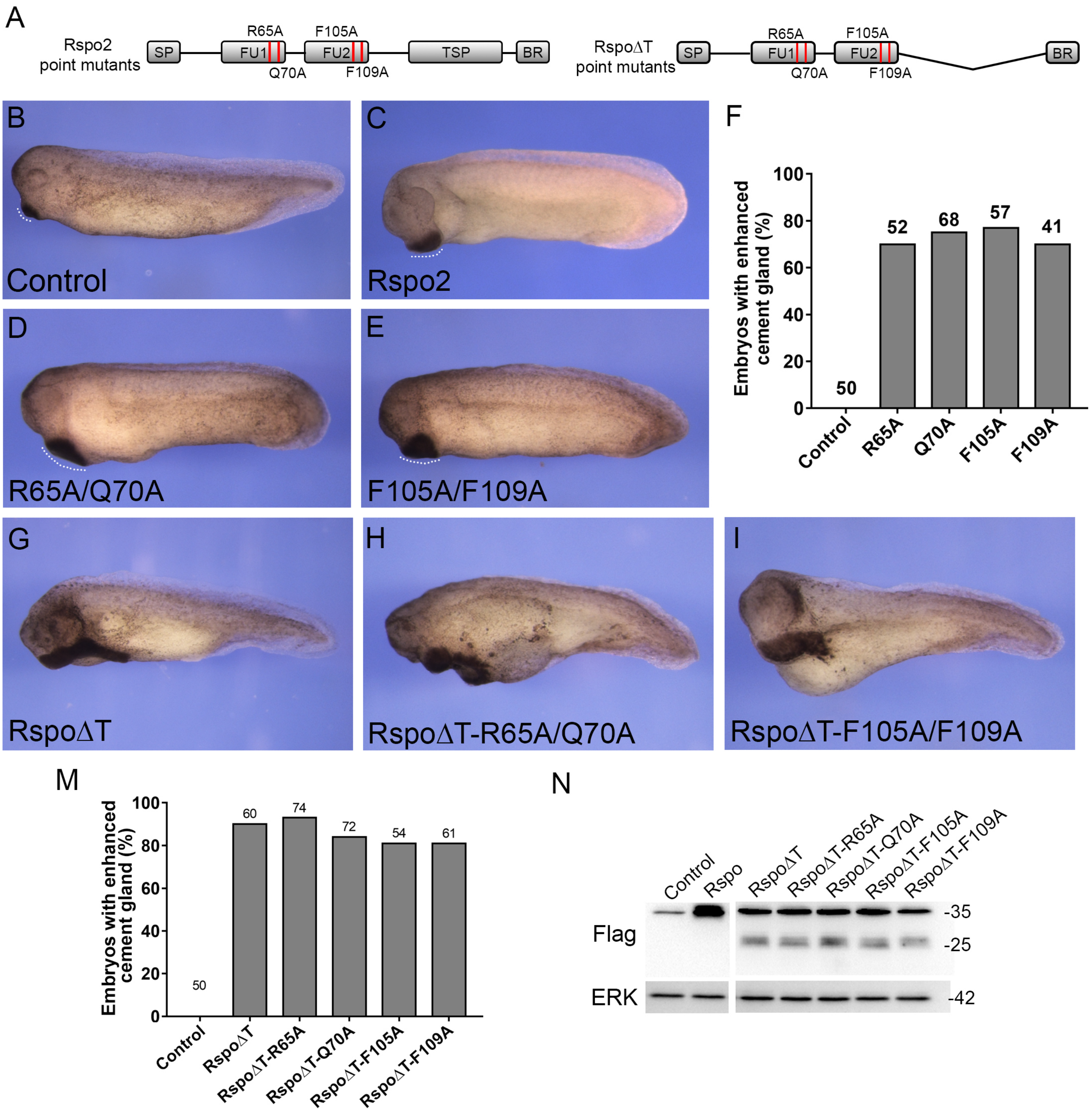
A
Rspo2
point mutants
SP
FU1
FU2
TSP
BR
R65A
Q70A
F105A
F109A
RspoΔT
point mutants
SP
FU1
FU2
BR
R65A
Q70A
F105A
F109A
B
Control
C
Rspo2
D
R65A/Q70A
E
F105A/F109A
F
Embryos with enhanced cement gland (%)
100
80
60
40
20
0
50
52
68
57
41
Control
R65A
Q70A
F105A
F109A
G
RspoΔT
H
RspoΔT-R65A/Q70A
I
RspoΔT-F105A/F109A
M
Embryos with enhanced cement gland (%)
100
80
60
40
20
0
50
60
74
72
54
61
Control
RspoΔT
RspoΔT-R65A
RspoΔT-Q70A
RspoΔT-F105A
RspoΔT-F109A
N
Control
Rspo
RspoΔT
RspoΔT-R65A
RspoΔT-Q70A
RspoΔT-F105A
RspoΔT-F109A
Flag
ERK
-35
-25
-42

Suppl. Fig 3

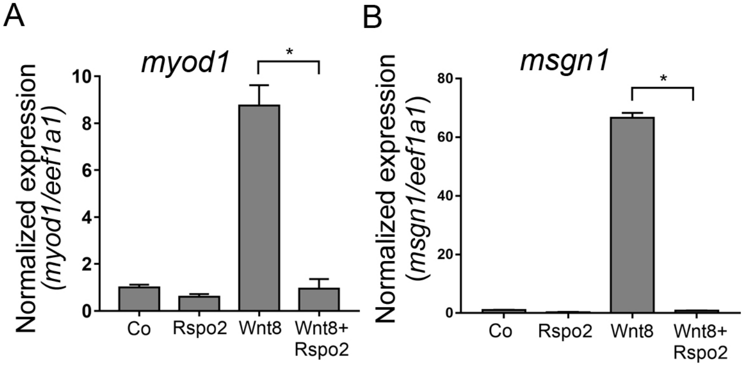

A
myod1
Normalized expression (myod1/eef1a1)
10
8
6
4
2
0
*
Co
Rspo2
Wnt8
Wnt8+ Rspo2
B
msgn1
Normalized expression (msgn1/eef1a1)
80
60
40
20
0
*
Co
Rspo2
Wnt8
Wnt8+ Rspo2

Suppl Fig. 4

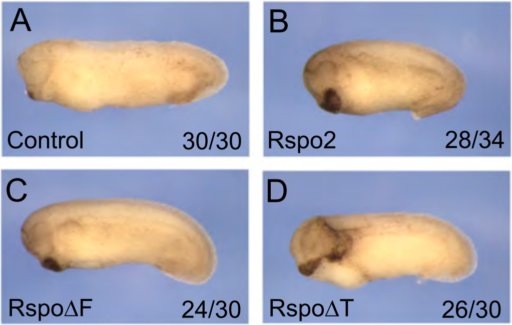

Suppl. Figure 5, relevant to Figure 3, Figure 4, Figure 5 and Supplementary Figure 2.

Original blots are shown, with the lanes included in Main Figures in red boxes.

Figure 3D (Lanes 1-4 are not relevant and not included in the main figure)

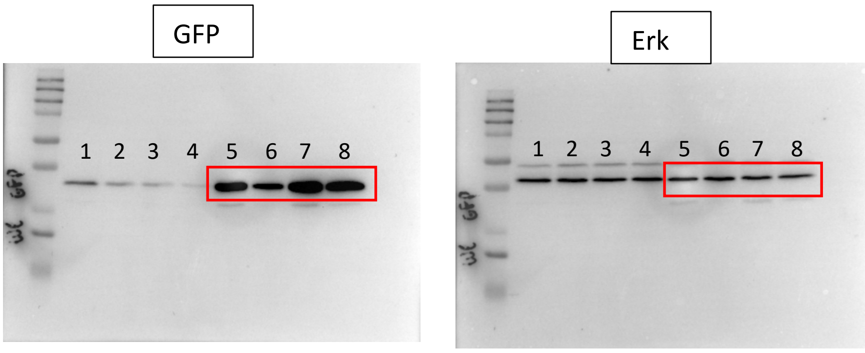

Figure 3E (Lanes 5 and 8 are not relevant and have not been included in the main figure)

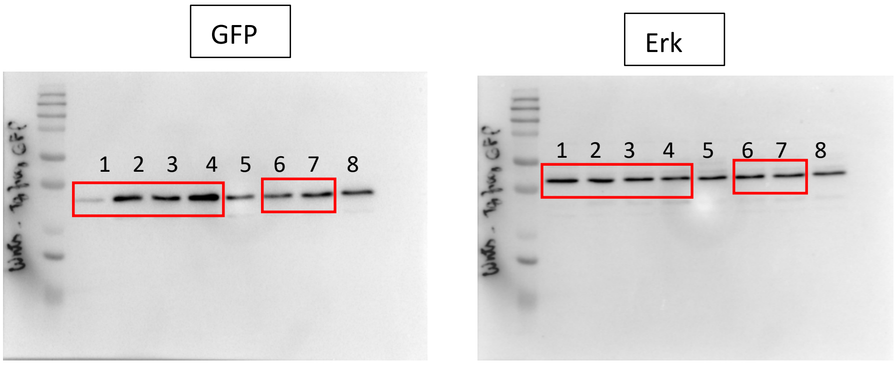

Figure 4B

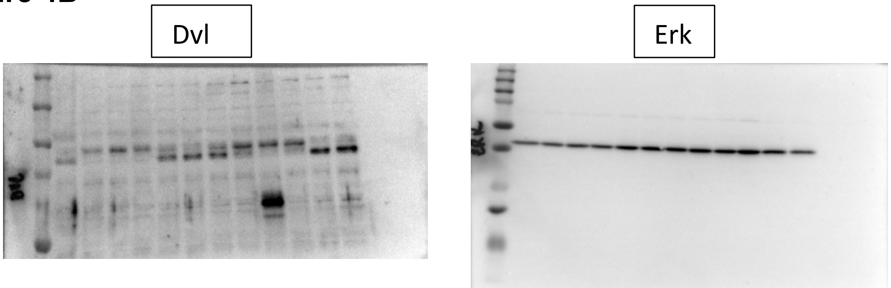

Figure 4C (the last lane is not relevant to the experiment and not shown in the main figure).

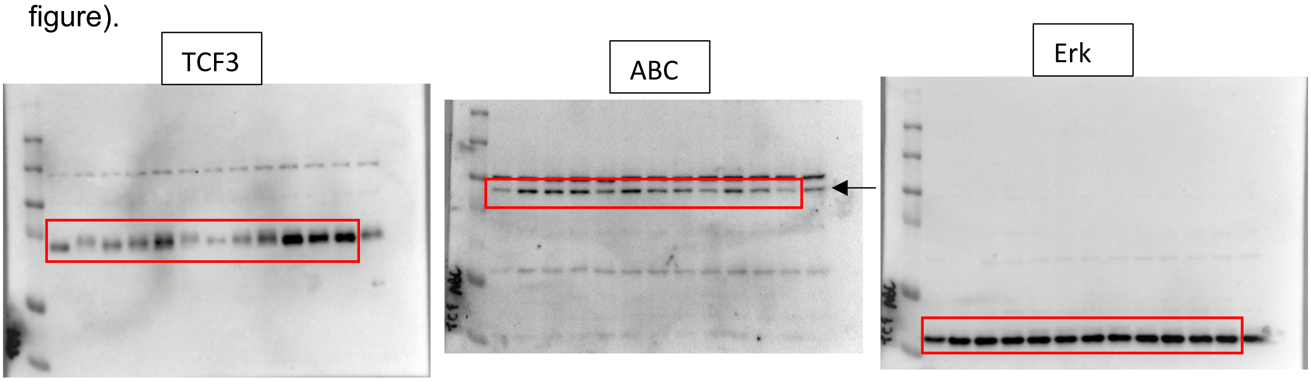

Figure 4D (lanes 3,4,8,9 are not relevant to the experiment and not included in the main figure).

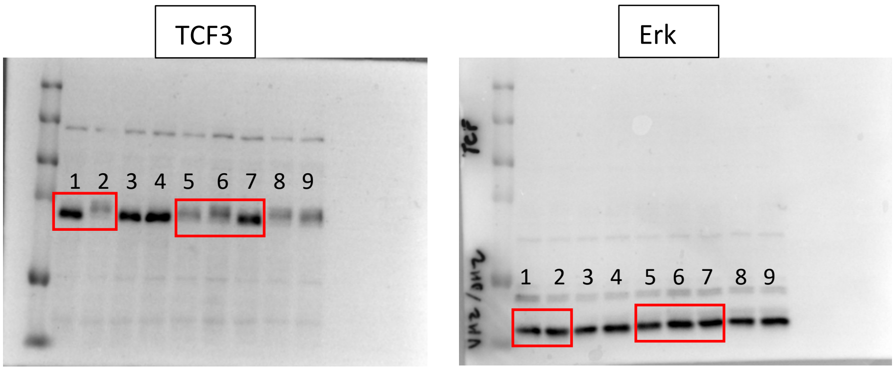

Figure 4E

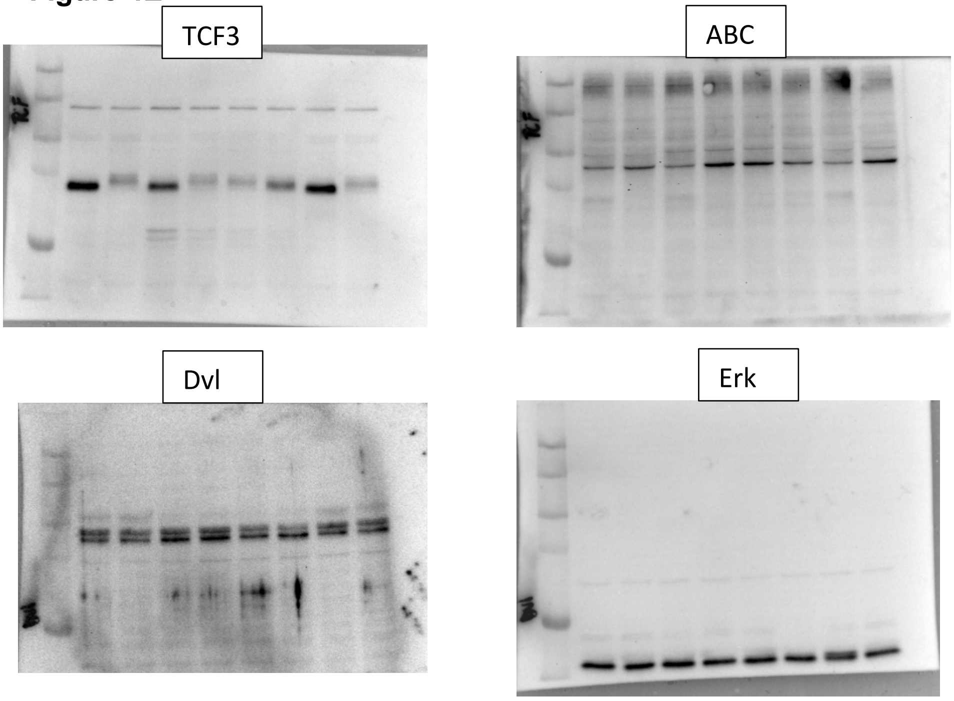

Figure 5C

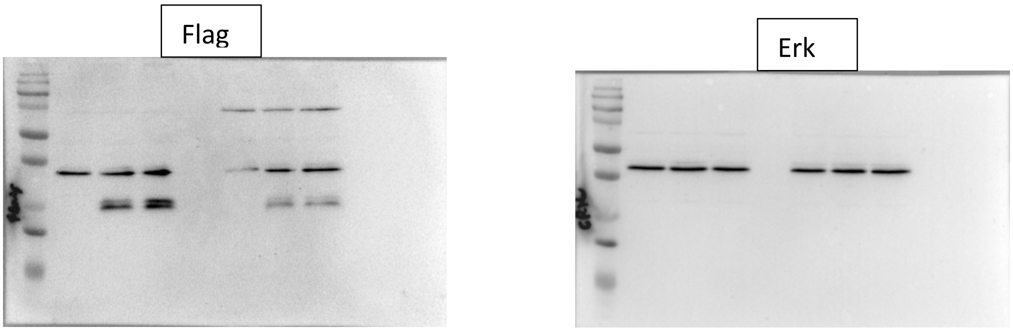

Supplementary Figure 2.

Lanes 1 and 2 have been rerun and shown at higher exposure and lane 3 is not relevant and not included.

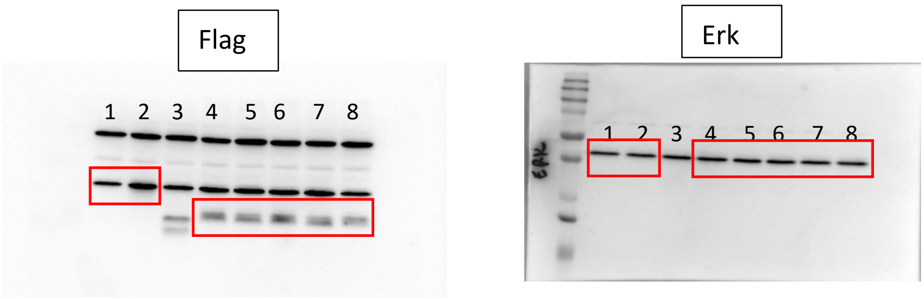

Supplementary Table 1

Quantification of wholemount *in situ* hybridization analysis in embryos with modulated Rspo2 levels

	**Control**	**Rspo2 RNA**	**RMO^{ATG}**
krt12.4			
Increased	0	0	13
Decreased	0	7	0
Unchanged	30	3	4
foxg1			
Increased	0	5	0
Decreased	0	0	12
Unchanged	22	7	3
cdx4			
Increased	3	0	12
Decreased	0	7	0
Unchanged	19	5	3

Supplementary Table 2.

Primers for Rspo2 mutagenesis

Rspo∆F:

5’- AGACGGAGCAAGAGAGCCAGATCTCCATTGGATGACACCATG-3’

Rspo∆T:

5’ -TGCGTGGATGGCTGTGAAGCTAGCGGAGGAACAAGAACCACA-3’

R65A:

5’- ACTGTTTTTCTATCTGCGCGCAGAAGGTATGAGGCAGTAT-3’

Q70A:

5’-CGAAGAGAAGGTATGAGGGCATATGGAGAGTGTCTGCAG-3’

F105A:

5’-GAAAATTGTGACTCTTGTGCATGCCGAGATTTTTGCATAA-3’

F109A:

5’-TCTTGTTTTAGCCGAGATGCATGCATAAAGTGCAAATCT-3’

Primers for RT-qPCR

otx2.L:	F: 5’-GGATGGATTTGTTACATCCGTC-3’
	R: 5’-CACTCTCCGAGCTCACTTCCC-3’
ag1.S:	F: 5’-GGTGCTGCCAAGTCTGAGC-3’
	R: 5’-GCCAGTTTCTGTGCCATTTTGTCA-3’
krt12.4.L:	F: 5’-CACCAGAACACAGAGTAC-3’
	R: 5’-CAACCTTCCCATCAACCA-3’
cdx4.L:	F: 5’-TGATTTATCACCTAACCAG-3’
	R: 5’-GTCCCAGATGGATGAGGAGA
msgn1.L:	F: 5’-GTATCCAACACTTTGCCATG-3’
	R: 5’-AGCACTGGAGAAGGTTTGTG-3’
axin-2-like:	F: 5’-GGCTGGTCTCTCTGCCTCTT-3’
	R: 5’-TGTCCTTCTCCTCCTGCTTCT-3’
eef1a1.S:	F: 5’-ACCCTCCTCTTGGTCGTTTT-3’
	R: 5’-TTTGGTTTTCGCTGCTTTCT-3’
myod1:	F: 5’-AGGTCCAACTGCTCCGACGGCATGAA-3’
	R: 5’-AGGAGAGAATCCAGTTGATGGAAACA-3’
